# Comparison of intravitreal bevacizumab to photodynamic therapy for polypoidal choroidal vasculopathy: Short-term results

**DOI:** 10.4103/0301-4738.64130

**Published:** 2010

**Authors:** Yoshinori Mitamura, Masayasu Kitahashi, Mariko Kubota-Taniai, Shuichi Yamamoto

**Affiliations:** 1 Department of Ophthalmology and Visual Science, Chiba University Graduate School of Medicine, Chiba, Japan; 2 Department of Ophthalmology, Institute of Health Biosciences, The University of Tokushima Graduate School, Tokushima, Japan

**Keywords:** Age-related macular degeneration, intravitreal bevacizumab, optical coherence tomography, photodynamic therapy, polypoidal choroidal vasculopathy

## Abstract

**Aims:**

To compare the short-term therapeutic effects of intravitreal bevacizumab (IVB) to those of photodynamic therapy (PDT) for polypoidal choroidal vasculopathy (PCV).

**Materials and Methods:**

Retrospective interventional case study. Eighty-nine eyes of 89 patients with symptomatic PCV were treated by IVB or PDT. Eighteen eyes were treated with a single injection of IVB (s-IVB group), 22 eyes with three consecutive monthly IVB injections (m-IVB group), and 49 eyes with PDT alone (PDT group). The best-corrected visual acuity (BCVA) and OCT-determined central foveal thickness (CFT) were evaluated before, and one and three months after the treatment. For statistical analyses, one-factor ANOVA and Chi-square test were used.

**Results:**

The differences in the BCVA and CFT among the three groups at the baseline were not significant (*P*=0.992, *P*=0.981, respectively). Three months after the treatment, the BCVA improved by >0.2 logMAR units in two out of 18 eyes (11%) in the s-IVB group, three out of 22 eyes (14%) in the m-IVB group, and 15 out of 49 eyes (31%) in the PDT group (*P*=0.124). A decrease in the CFT by >20% was achieved in six out of 18 eyes in the s-IVB group, ten eyes (46%) in the m-IVB group, and 35 eyes (71%) in the PDT group (*P*=0.009). The resolution of polyps was achieved in three out of 18 eyes in the s-IVB group, one eye (5%) in the m-IVB group and 35 eyes (71%) in the PDT group (*P*<0.001).

**Conclusion:**

The better short-term therapeutic outcomes in the PDT group than in the s-IVB and m-IVB groups indicate that PDT may be more effective than IVB in short term after treatment for PCV.

Idiopathic polypoidal choroidal vasculopathy (PCV), first described by Yannuzzi *et al*,[1] appears as reddish-orange lesions that are polypoidal dilations of the choroidal vascular network. PCV frequently leads to an insidious decrease of visual acuity due to serosanguinous complications affecting the macula,[2] but some eyes with PCV may remain clinically silent with no leakage, i.e. asymptomatic polyps. Occasionally, eyes with PCV have an acute and severe loss of vision secondary to massive submacular or vitreous hemorrhage from spontaneously ruptured vessels.

The incidence of PCV is higher in Japanese and other Asian populations than in the Caucasian populations. The prevalence of PCV was reported to be 54.7% in Japanese patients with neovascular age-related macular degeneration (AMD).[3]

It has been reported that photodynamic therapy (PDT) with verteporfin is effective for treating PCV with subfoveal involvement.[2,4‐8] The responsiveness of a PCV to PDT may be because it shares some similarities in its clinical properties and histology with the choroidal neovascularization (CNV) of eyes with AMD. It has been reported that PDT is more effective than transpupillary thermotherapy for the treatment of eyes with PCV.[9] PDT has led to good results for PCV, but extensive subretinal hemorrhage is an unavoidable side-effect of PDT in some cases.[2,6] It has been reported that a recurrence of polyps is another complication that reduces visual acuity after PDT.[7]

It has been reported that the aqueous humor level of vascular endothelial growth factor (VEGF) was higher in eyes with PCV than in eyes with AMD,[10] suggesting an association between VEGF and PCV. Bevacizumab (Genentech Inc., South San Francisco, CA, USA), a humanized monoclonal antibody that inhibits all VEGF isoforms, has shown promising results against CNVs that were secondary to AMD.[11‐14] Gomi *et al*.,[15] reported that intravitreal bevacizumab (IVB) was effective in reducing the fluid from PCV but not for diminishing choroidal vascular changes. In addition, they reported that a single IVB was insufficient for the treatment of PCV and that regular injections might maintain vision over a longer time because of the anti-leakage effect of bevacizumab on the exudative changes due to PCV.[15]

To date, a direct comparison of the therapeutic efficacy of IVB for PCV to that of PDT has not been reported. The purpose of this study was to compare the short-term therapeutic effects of IVB to those of PDT for eyes with PCV.

## Materials and Methods

retrospective This interventional case study was done on 89 eyes of 89 consecutive Japanese patients who had treatment-naїve PCV and subfoveal exudation or hemorrhage, and were treated with IVB or PDT with verteporfin. The diagnosis of PCV was based on clinical examination, fluorescein angiography (FA), and indocyanine green angiography (ICGA). The criteria for a diagnosis of PCV were the presence of reddish-orange lesions, recurrent serosanguinous retinal pigment epithelium (RPE) detachments, and dilated network of inner choroidal vessels with terminal hyperfluorescent aneurysm-like dilatations (polyps) on ICGA. A diagnosis for PCV was made only in the presence of the ICGA features. None of the cases had a secondary CNV on clinical examination or FA. The procedures used conformed to the tenets of the Declaration of Helsinki, and an approval was obtained from the Institutional Review Board. An informed consent was obtained from all patients.

Among the 89 eyes, 49 eyes were treated with PDT alone (PDT group) between June 2004 and July 2006, 18 eyes with a single IVB (s-IVB group) between August 2006 and July 2007, and 22 eyes with three consecutive monthly IVB (m-IVB group) between August 2007 and August 2008. In our institute, monthly IVB could not be performed between August 2006 and July 2007, because the Institutional Review Board initially required a three-month observational period after IVB to detect the side-effect of IVB. All eyes undergoing treatment for PCV between June 2004 and July 2007 were included in this study. After the three-month follow-up period, some eyes in the s-IVB and m-IVB groups were retreated with PDT or IVB. Only symptomatic patients with PCV and visual disturbance due to subfoveal exudation or hemorrhage were treated in the PDT, s-IVB and m-IVB groups. There was no difference in terms of treatment indication among the three groups. There were one woman and 17 men in the s-IVB group, five women and 17 men in the m-IVB group, and seven women and 42 men in the PDT group (*P* = 0.309; Chi-square test; Table 1). The mean age at presentation was 72.9 ± 5.7 (± standard deviation) years in the s-IVB group, 73.0 ± 8.9 years in the m-IVB group and 69.6 ± 7.8 years in the PDT group (*P* = 0.131, Chi-square test).

**Table 1:** Pretreatment characteristics of the s-IVB, m-IVB and PDT groups

	s-IVB group	m-IVB group	PDT group	*P* value
Number of eyes	18	22	49	
Women/Men	1/17	5/17	7/42	0.309
Age (years)	72.9±5.7	73.0±8.9	69.6±7.8	0.131
BCVA (logMAR units)	0.54±0.37	0.53±0.34	0.54±0.29	0.992
Foveal thickness (μm)	427.9±190.2	438.8±234.7	438.5±192.3	0.981
GLD (μm)	3366±992	3651±1833	3718±1665	0.726

s-IVB: Single intravitreal bevacizumab, m-IVB: Monthly intravitreal bevacizumab, PDT: Photodynamic therapy, BCVA: Best-corrected visual acuity, GLD: Greatest linear dimension

The bevacizumab was prepared by the institutional pharmacy as sterile filled and packed tuberculin syringes containing 0.1 ml. The intravitreal injection of 1.25 mg/0.05 ml bevacizumab was carried out with a 30-gauge needle 3.0-4.0 mm posterior to the limbus after topical anesthesia.

PDT with verteporfin was performed according to the guidelines of the Treatment of Age-Related Macular Degeneration with Photodynamic Therapy (TAP) Study.[16] Five minutes after the completion of a standard verteporfin infusion, the laser beam at 689 nm was given.

Silva *et al*.,[4] reported that the ICGA hotspots would probably indicate the size of the PCV lesions to be treated with PDT. For this study, the size of the laser spot for PDT was chosen to cover the polyps and the surrounding abnormally dilated choroidal vessels seen on ICGA plus an additional 1000-μm margin.[2,4,5] Three months after treatment, ICGA was performed to examine the resolution of polyps in all the 89 eyes.

In the s-IVB and PDT groups, re-treatment was not performed during the three months after the initial treatment. On the other hand, all eyes completed three-monthly injections of bevacizumab in the m-IVB group.

The best-corrected visual acuity (BCVA) was measured with a Japanese standard Landolt visual acuity chart and converted to the logarithm of the minimal angle resolution (logMAR) units. The BCVA was measured before, and one and three months after the treatment. A significant improvement or decrease of the visual acuity was defined as a change of >0.2 logMAR units. The central foveal thickness (CFT) was measured by optical coherence tomography (OCT; Stratus III OCT, Carl Zeiss, Dublin, CA, USA) using five-mm scans before, and one and three months after treatment. The CFT was measured by placing calibrated calipers at the vitreous-retina interface and presumed inner border of the RPE, and included the thickness of subretinal fluid. When the fixation was poor, scans were centered on the fovea under video observation. An improvement of foveal thickness was defined as >20% decrease in foveal thickness.

The significance of the differences of age, BCVA, CFT and the greatest linear dimension (GLD) in the three groups was tested statistically using one-factor ANOVA. The significance of the differences of gender and polyp location in the three groups was tested statistically using Chi-square test. To determine the significance of the changes among the three groups, paired t tests for continuous variables and Chi-square test or Fisher exact probability test for categorical variables were used. The correlations among changes in the BCVA, decrease in the CFT and the resolution of polyps were determined by t test or Pearson's correlation tests. The level of statistical significance was set at *P* <0.05.

## Results

**Figure 1:**
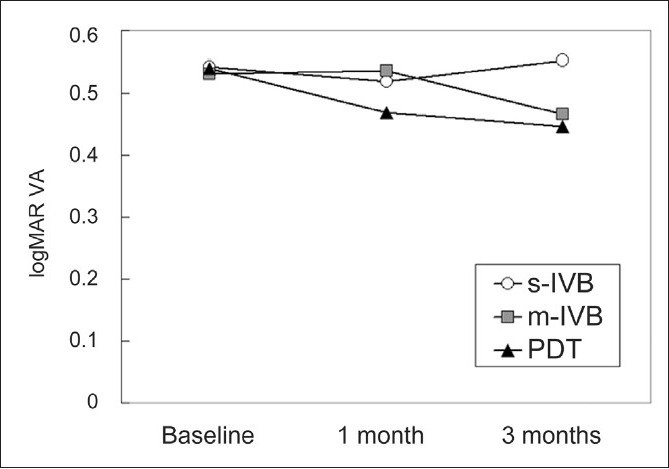
Comparison of logMAR visual acuities among the single intravitreal bevacizumab (s-IVB) group, the monthly intravitreal bevacizumab (m-IVB) group and the PDT group after treatment

At the baseline, the GLD was 3366±992 μm in the s-IVB group, 3651±1833 μm in the m-IVB group, and 3718±1665 μm in the PDT group. These differences among the three groups were not significant (*P*=0.726, one-factor ANOVA; Table 1). As for the location of polyps, no eye had polyps around the disc in the s-IVB, m-IVB and PDT groups. Polyps were located in the submacular area in 11 eyes of the s-IVB group, 14 eyes of the m-IVB group and 26 eyes of the PDT group. In the other eyes of each group, polyps were located around the macula. There was no significant difference in the location of polyps among the three groups (*P*=0.661, Chi-square test). The differences in the BCVA among the three groups at the baseline were not significant (*P*=0.992, one-factor ANOVA; Table 1). Three months after treatment, there was no significant difference in the BCVA among the three groups (*P*=0.553, one-factor ANOVA; Table 2). However, the BCVA at one or three months after treatment was significantly better than the baseline BCVA in the PDT group (*P*=0.004, *P*=0.002, respectively, paired t tests; Fig. 1). At one and three months, there was no significant change in the BCVA in the s-IVB (*P*=0.535, *P*=0.795, respectively) and m-IVB groups (*P*=0.844, *P*=0.152, respectively, paired t test).

**Table:** 

	Baseline	1 month	3 months
s-IVB	0.541974178	0.518501500	0.551949277
m-IVB	0.530743361	0.536733278	0.466237484
PDT	0.540489713	0.468231406	0.446509501

**Table 2:** Treatment results of the s-IVB, m-IVB and PDT groups at three months after treatment

	s-IVB group	m-IVB group	PDT group	*P* value
BCVA (logMAR)	0.55±0.37	0.47±0.37	0.45±0.34	0.553
Improvement of BCVA (>0.2)	Two out of 18 eyes	Three eyes (14%)	15 eyes (31%)	0.124
Foveal thickness (μm)	353.3±162.3	331.5±194.0	269.9±157.8	0.133
Improvement of foveal thickness (>0.20%)	Six out of 18 eyes	10 eyes (46%)	35 eyes (71%)	0.009
Resolution of polyps	Three out of 18 eyes	One eye (5%)	35 eyes (71%)	<0.001

s-IVB: Single intravitreal bevacizumab, m-IVB: Monthly intravitreal bevacizumab, PDT: Photodynamic therapy, BCVA: Best-corrected visual acuity

In the s-IVB group, the BCVA improved by >0.2 logMAR units in two out of 18 eyes, remained unchanged in 14 eyes, and worsened in two eyes at three months after treatment [Table 2]. In the m-IVB group, the BCVA improved by >0.2 logMAR units in three eyes (14%), remained unchanged in 17 eyes (77%), and worsened in two eyes (9%). In the PDT group, the BCVA improved by >0.2 logMAR units in 15 eyes (31%), remained unchanged in 30 eyes (61%), and worsened in four eyes (8%). There was no significant difference in the number of eyes whose BCVA improved by >0.2 logMAR units among the three groups at three months after treatment (*P*=0.124, Chi-square test; Table 2). However, the BCVA improvement tended to be better in the PDT group.

**Figure 2:**
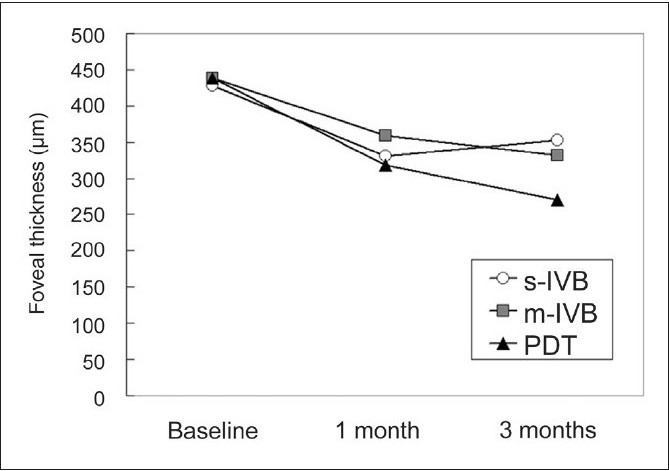
Comparison of OCT-determined foveal thickness among the single intravitreal bevacizumab (s-IVB), monthly intravitreal bevacizumab (m-IVB) and PDT groups after treatment

There was no significant difference in the pre-treatment CFT among the three groups (*P*=0.981, one-factor ANOVA; Table 1). Three months after treatment, there was also no significant difference in the CFT among the three groups (*P*=0.133, one-factor ANOVA; Table 2). The fovea was significantly thinner at one or three months after treatment than at the baseline in the s-IVB (*P*=0.003, *P*=0.021, respectively), m-IVB (*P*=0.018, *P*=0.023, respectively) and PDT groups (both *P*<0.001, paired t test; Fig. 2). In the s-IVB group, the CFT tended to increase at three months after treatment compared with that at one month, although the difference was not significant (*P*=0.077, paired t test; Fig. 2).

**Table:** 

	Baseline	1 month	3 months
s-IVB	427.944444	331.222222	353.333333
m-IVB	438.772727	358.681818	331.454546
PDT	438.469388	318.367347	269.938776

In the s-IVB group, the CFT decreased by >20% in six out of 18 eyes, remained unchanged in 11 eyes, and worsened in one eye [Table 2] at three months after treatment. In the m-IVB group, the CFT decreased by >20% in ten eyes (46%), remained unchanged in nine eyes (41%), and worsened in three eyes (14%). In the PDT group, the CFT decreased by >20% in 35 eyes (71%), remained unchanged in 14 eyes (29%), and worsened in 0 eye (0%). There was a significant difference in the number of eyes whose CFT decreased by >20% among the three groups at three months after treatment (*P*=0.009, Chi-square test; Table 2). When the incidence of eyes whose CFT decreased by >20% in the three groups was compared independently with each of the other groups, a decrease in the CFT was found significantly more frequently in the PDT group than in the s-IVB (*P*=0.005) and m-IVB groups (*P*=0.037, Chi-square test). However, there was no significant difference between the s-IVB and m-IVB groups (*P*=0.436, Chi-square test).

**Figure 3:**
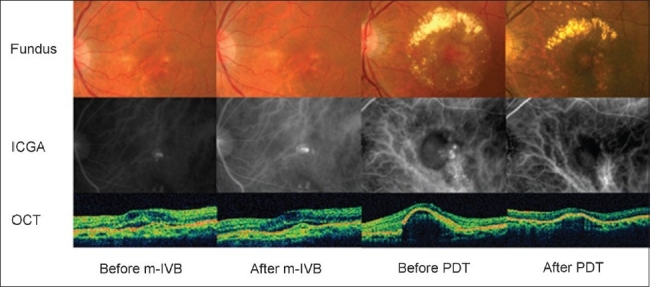
Fundus photograph, indocyanine green angiographic (ICGA) and OCT images in representative cases treated by monthly intravitreal bevacizumab (m-IVB) or PDT. After m-IVB, the terminal hyperfluorescent aneurysm-like dilatations (polyps) are still present in the ICGA images. However, polyps disappear after PDT

Three months after treatment, a resolution of polyps on ICGA was achieved in three out of 18 eyes in the s-IVB group, one eye (5%) in the m-IVB group, and 35 eyes (71%) in the PDT group (*P*<0.001, Chi-square test; Table 2). When the polyp resolution of the three groups was compared independently with each of the other groups, polyp resolution was significantly more frequent in the PDT group than in the s-IVB and m-IVB groups (both *P*<0.001, Fisher exact probability test). However, there was no significant difference between the s-IVB and m-IVB groups (*P*=0.310, Fisher exact probability test). Representative cases of the m-IVB and PDT groups are presented in Fig. 3. Branch vascular network on ICGA had not changed in all eyes of s-IVB, m-IVB and PDT groups.

In all the 89 eyes in the s-IVB, m-IVB and PDT groups, the correlations among changes in the BCVA, decrease in the CFT and the resolution of polyps on ICGA were examined. The change in the BCVA was defined as the logMAR BCVA at baseline subtracted from the logMAR BCVA three months after the treatment. The decrease in the CFT was defined as a ratio of the change in the CFT from the baseline divided by the CFT at the baseline. The resolution of polyps was achieved in 39 of the 89 eyes. The change in the BCVA was significantly better in the eyes with the resolution of polyps (-0.114±0.201) than in those without (-0.028±0.185) (*P*=0.039, t test). The decrease in the CFT was significantly better in the eyes with the resolution of polyps (0.399±0.225) than in those without (0.171±0.294) (*P*<0.001, t test). The changes in the BCVA was not significantly correlated with the decrease in the CFT (r = 0.174, *P*=0.103; Pearson's correlation tests).

In the s-IVB and m-IVB groups, none of the patients developed systemic complications related to IVB, including thromboembolic events or cerebral vascular accidents. Ocular complications such as intraocular inflammation, cataract progression and endophthalmitis were also not found. In the PDT group, no complications related to PDT, including RPE tears, photosensitivity, low back pain and catheter-induced complications, were observed.

## Discussion

Our investigations showed that the resolution of polyps and improvement of the BCVA and CFT were found more frequently in the PDT group than in the s-IVB and m-IVB groups during the three-month follow-up period. The BCVA improved significantly in the PDT group but not in the s-IVB and m-IVB groups. The CFT improved significantly not only in the PDT group but also in the s-IVB and m-IVB groups. Although our results are limited by the short follow-up period and non-randomized nature, our observations indicate that both PDT and IVB have beneficial effects on eyes with PCV, but the therapeutic outcomes are better with PDT. In the s-IVB group, the CFT tended to increase at three months after treatment compared with that at one month. Song *et al*.,[17] also reported that the CFT increased slightly at three months from the one-month level after a single IVB. Therefore, a single injection of bevacizumab is not sufficient in treating PCV, although the IVB can reduce the fluid from PCV.

The best treatment for PCV has still not been established. A conservative approach is recommended unless visual acuity is decreased because of progressive exudative changes or an acute submacular hemorrhage. PDT is effective for treating AMD, and a number of studies have demonstrated its efficacy in treating PCV.[2,4‐8] Chan *et al*.,[2] reported that the visual acuity was stable or improved in 95% (21/22) eyes at the one-year follow-up, and Silva *et al*.,[4] reported that the BCVA improved or stabilized in 17 of 21 (81%) eyes after one year.

Gomi *et al*.,[15] reported that the polyps were resolved on ICGA in only one out of 11 eyes, unchanged in eight eyes, and increased in two eyes with PCV after IVB. They reported that they could not determine whether additional IVB was effective in their case series. Thereafter, Lai *et al*.,[18] reported that polyps persisted in all 15 eyes at three months after three monthly IVB. However, three eyes with a previous PDT were included. Our study group included only treatment-naїve eyes, and a resolution of the polyps was achieved only in three out of 18 eyes after a single IVB and in one eye (5%) after three monthly IVB. Taken together, these results indicate that IVB has limited effectiveness in the regression of the polyps. In our study, a resolution of polyps was achieved in 35 eyes (71%) after PDT. Therefore, PDT is more effective than IVB for the resolution of polyps.

Bevacizumab is a full-length humanized monoclonal antibody for VEGF and a relatively large molecule. Recently, it was reported that bevacizumab was nontoxic to the retina of rabbits based on electrophysiological studies, and that bevacizumab passed through the full thickness of the retina at 24 h and was essentially absent at four weeks.[19] However, the polyps and choroidal vascular network of PCV are located below the RPE. Pedersen *et al*.,[20] reported that AMD patients with RPE detachments had a significantly worse BCVA after IVB. Limited penetration of bevacizumab into the sub-RPE space might lead to relatively worse outcomes of IVB for PCV compared with that of PDT. Gomi *et al*.,[15] reported that one eye with complete resolution of polyps after IVB had an atrophic RPE, and suggested that bevacizumab might have reached the sub-RPE polyps in sufficient concentration through the atrophic RPE. In this study, however, RPE atrophy was not found in eyes with resolution of polyps after IVB.

The CFT significantly decreased not only in the PDT group but also in the s-IVB and m-IVB groups. Because IVB monotherapy reduces exudative changes, IVB might have a role in combination therapy with PDT.[18] In AMD, increased expression of VEGF has been reported in the CNV of eyes after PDT.[21] This elevated expression of VEGF following PDT might potentially increase the risk of CNV recurrences.[21] Similarly, the addition of IVB to PDT for PCV might counteract the up-regulation of VEGF following PDT and might prevent the recurrence of polyps. Pai *et al*.,[22] reported that one case with PCV and CNV was successfully treated with IVB followed by PDT. Lee *et al*.,[23] reported that the BCVA improved by ≥two lines in seven out of 12 eyes treated with IVB alone or in combination with PDT. In their study, however, five eyes had a history of treatment, and the number and interval of IVB treatment were different in each case. Thus, further studies to evaluate the synergistic effect of IVB and PDT for the treatment for PCV are needed.

Our results suggest that PDT may be more effective than IVB monotherapy in short term after treatment for PCV. However, our study was non-randomized and the follow-up period was short. In this study, PDT was performed in some eyes of the s-IVB and m-IVB groups after the three-month follow-up period. Therefore, we could not examine the results after the three-month follow-up period. To exactly compare the effectiveness of PDT and IVB, a randomized longitudinal study is necessary. In addition, further studies to evaluate the efficacy of other anti-VEGF agents and combination therapy of PDT and IVB for PCV are required.
